# Genetic and Epigenetic Approaches for the Possible Detection of Adulteration and Auto-Adulteration in Saffron (*Crocus sativus* L.) Spice

**DOI:** 10.3390/molecules21030343

**Published:** 2016-03-11

**Authors:** Giovanna Soffritti, Matteo Busconi, Rosa Ana Sánchez, Jean-Marie Thiercelin, Moschos Polissiou, Marta Roldán, José Antonio Fernández

**Affiliations:** 1 Department of Sustainable Crop Production, Faculty of Agriculture, Food and Environmental Sciences, Università Cattolica del Sacro Cuore, Via Emilia Parmense 84, Piacenza 29122, Italy; giovannasoffritti@alice.it; 2 BioDNA, Centro di Ricerca sulla biodiversità e sul DNA antico, Università Cattolica del Sacro Cuore, Piacenza 29122, Italy; 3 Laboratory of Biotechnology and Natural Resources, Institute for Regional Development (IDR), Universidad de Castilla-La Mancha, IDR-Biotecnología, Campus Universitario s/n, Albacete 02071, Spain; rosanasanz@hotmail.com (R.A.S.); marta.roldan@uclm.es (M.R.); 4 Tradimpex Jm Thiercelin sas, Parc de l’Ecopôle 3 Rue Pierre et Marie Curie, Combs La Ville 77380, France; jean@thiercelin.com; 5 Laboratory of Chemistry, Department of Food Science and Human Nutrition, School of Food, Biotechnology and Development, Agricultural University of Athens, 75 Iera Odos, Athens 11855, Greece; mopol@aua.gr

**Keywords:** saffron, adulteration, DNA-based traceability, molecular markers

## Abstract

Saffron (*Crocus sativus* L.) is very expensive and, because of this, often subject to adulteration. Modern genetic fingerprinting techniques are an alternative low cost technology to the existing chemical techniques, which are used to control the purity of food products. *Buddleja officinalis* Maxim, *Gardenia jasminoides* Ellis, *Curcuma longa* L., *Carthamus tinctorius* L. and *Calendula officinalis* L. are among the most frequently-used adulterants in saffron spice. Three commercial kits were compared concerning the ability to recover PCR-grade DNA from saffron, truly adulterated samples and possible adulterants, with a clear difference among them, mainly with the processed samples. Only one of the three kits was able to obtain amplifiable DNA from almost all of the samples, with the exception of extracts. On the recovered DNA, new markers were developed based on the sequence of the plastid genes *matK* and *rbcL*. These primers, mainly those developed on *matK*, were able to recognize saffron and the adulterant species and also in mixtures with very low percentages of adulterant. Finally, considering that the addition of different parts of saffron flowers is one of the most widespread adulterations, by analyzing the DNA of the different parts of the flower (styles, stamens and tepals) at the genetic and epigenetic level, we succeeded in finding differences between the three tissues that can be further evaluated for a possible detection of the kind of fraud.

## 1. Introduction

Saffron is an ancient spice that consists of the dehydrated stigmas of the triploid sterile plant *Crocus sativus* Linn. [1]. The spice, other than for culinary uses and colorant properties, contains different secondary metabolites, and because of this, it has been historically used in traditional medicine for a number of health properties, many of which have been scientifically confirmed or supported in studies reported in a recent review [2]. Saffron is renowned as the most expensive spice; its market price ranks among the highest in foods, reaching 20,000 €/kg and more for some PDO (Protected Designation of Origin) productions in 2015, and it is the highest priced high value agricultural product (HVAP) in the world. Saffron can be found on the market in the form of entire dried stigmas or as a finely-ground powder. The high price is a consequence of the high manual labor required for its cultivation, harvesting and handling: approximately 150,000 flowers must be carefully picked one by one in order to produce 1 kg of the spice. Among the major candidates for adulteration, saffron is one of the most targeted foods and spices. As a consequence, adulteration represents a real and major concern for the saffron market, and such practice is more often performed in ground stigmas, where extraneous material can be more easily concealed [3]. Over the years, different adulterations have been detected, involving the addition of different plant species, animal-derived substances, synthetic dyes and chalk, among others [4,5]. Nowadays, topics such as food authenticity, genuineness and the detection of adulteration in food products, usually economically motivated, are increasingly important for consumers, regulatory agencies and the food industry [6] Presently, within the most-frequently reported plant materials used to adulterate saffron, there are: (1) cut and/or dyed *C. sativus* stamens; (2) safflower and calendula petals (*Carthamus tinctorius* L. and *Calendula officinalis* L.); (3) curcuma powdered rhizomes (*Curcuma longa* L.); (4) gardenia yellow from *Gardenia jasminoides* Ellis fruits; and (5) dye extracted from the flowers of *Buddleja officinalis* Maxim. Additionally, commercial safflower and curcuma are often mislabeled, using the name “saffron” and the supposed country of origin to mislead consumers. For the detection of plant adulterants in saffron, several chromatographic methods, reported in [6], and the use of nuclear magnetic resonance (NMR) spectroscopy [6] have provided interesting results. In addition to these methods, modern genetic fingerprinting techniques are alternative low cost technologies that enable the identification of individual plant material in raw matrices and in processed food, once proper methods for adequate DNA extraction and amplification are available [3]. These methodologies are highly specific, require very small amounts of sample for analysis (potentially even micrograms) and are considered an ideal technology with which to implement the existing methods in controlling the purity of food products. The development of molecular markers for saffron traceability has been recently addressed [3,7,8]. In a recent paper [9], a method based on a barcoding melting curve analysis using the universal chloroplast plant DNA barcoding region *trnH-psbA* was developed for the detection of adulterants in traded saffron. In order to continue implementation of the existing methodologies to detect the presence of the main plant adulterants in saffron production, the present work has been focused on the comparison of different DNA extraction methods to better recover DNA from the considered matrices and on the development of DNA markers to identify the presence, in saffron production, of adulterants, such as *Buddleja officinalis*, *Gardenia jasminoides*, *Curcuma longa*, *Carthamus tinctorius* and *Calendula officinalis*. Liquid and solid extracts of gardenia and buddleia have been considered, as well. 

At the molecular level, DNA methylation of cytosine with the conversion to 5-methylcytosine is one of the most widespread epigenetic modifications [10]. While the DNA sequence is conserved among the different tissues of the organism, the methylation state of cytosines can change, influencing chromatin structure and gene expression. Analysis of the methylation pattern can be used to differentiate between different tissues. Consequently, considering that adulteration can also be carried out by adding different parts of the crocus flower itself, the comparison of the MS-AFLP (methyl-sensitive-amplified fragment length polymorphism) and AFLP (amplified fragment length polymorphism) profiles of tepals, stamens and stigmas has been carried out in order to show that polymorphic signals are potentially useful for traceability purposes.

## 2. Results and Discussion

### 2.1. DNA Extraction and PCR Amplificability

The recovery of DNA from complex matrices like plant-derived food matrices is of fundamental importance to establish reliable methods for DNA-based food traceability. Several obstacles must be faced when working with DNA from food and processed samples, in particular: the presence of inhibitors (secondary metabolites) that can hinder enzymatic reactions, such as PCR [11,12], and the DNA that is usually recoverable from these matrices can be very poor and highly degraded [13]. If the DNA is highly degraded, it may not be available to the polymerases, which are stopped by sites of damage, meaning that the reaction may be interrupted, which can influence the length and significance of the synthesized amplicons. In this study, we compared three different kits to identify the most suitable one for our aim. After the extraction of DNA from the available samples (Table 1), we found that, with the exception of some samples (mainly leaves, tepals, stamens and stigmas), all of the remaining samples were characterized by the presence of very weak smears or by the absence of any signals. Concerning the results of the DNA extractions, no significant differences were evident among the three kits in terms of presence/absence and intensity of the signal that was visible on agarose gel.

The presence of high DNA degradation hinders a correct quantification of the extraction, and when the signal is absent, it is almost impossible to correctly quantify the recovered DNA. In similar situations, the assembly of PCR reactions is usually considered the best way to check for the presence of any DNA and the only way to see whether the eventually present DNA is free of inhibitors.

For the PCRs, some universal markers for plant barcoding were used (Table 2). The application of such markers for the traceability of minor crops as spices has been recently reviewed [14], where it was reported that plant barcoding was applied to spices, such as oregano, sage, thyme and rosemary.

The PCRs were carried out with the addition of PVP in the amplification mix, because previous experiments, carried out by us on different food matrices, showed an improvement in DNA amplification after the addition of this substance.

The results of the amplification tests are reported in Table 3, and it was clear that with the GeneElute kit, it was possible to obtain the amplification in much more samples than with the other two methods that provided substantially the same results.

Some samples, the ones corresponding to petals, tepals and leaves (AD.04.POL, AD.06.POL, AD.18.BM, AD.19.BM, AD.20.BM, AD.21.BM and the samples for MS-AFLP and AFLP analysis) provided similar results with the three kits.

The samples AD.03.JM, AD.05.POL, AD.09.POL, AD.10.POL and AD.14.JM, corresponding to the solid or liquid extracts from gardenia and buddleia, did not amplify despite different extractions or amplification parameters. This may be a consequence of: a complete absence of DNA in the starting matrices; the inability to recover the DNA present; or the inability to amplify the recovered DNA because of inhibitors of the polymerase that were co-extracted along with DNA. In order to test the possible presence of inhibitors in the extracts, we prepared some mixtures of saffron and extracts in equal percentages (50% and 50%) and repeated both the DNA extraction and amplification using primers specific for saffron and the species of the extracts (gardenia and buddleia). By doing this, we always amplified just the crocus DNA, but never the other DNA. We could therefore exclude the presence of free inhibitors in the DNA suspension recovered from the mixture that can hinder the functionality of the polymerase and the amplification of saffron DNA. Because of this, we speculated that the most likely explanation is the absence of recoverable DNA in the extracts. We do not know how these extracts were obtained and what kind of treatment was applied to produce them. We cannot exclude the possible presence of inhibitors directly linked to the DNA of the extracts that can hinder its amplification [15].

For the remaining samples, the GeneElute Plant Genomic DNA Miniprep Kit gave the best performance; in fact, the PCR amplifications obtained using the DNA recovered with this kit were always intense. Based on these results, the DNAs used for the subsequent analyses were the ones recovered with the GeneElute kit. 

Another important aspect correlated with the extraction and use of food DNA is the maximum size of fragment that can be amplified via PCR. In fact, for processed food, also according to the intensity of the processing, the maximum amplicon size is not large and generally smaller than 500 bps. Despite this, for all of the amplifiable samples, we obtained amplicons with all three primer pairs, and interestingly, using the primer for plastid *matK*, we were able to amplify fragments larger than 900 bps. It is generally considered that plastid DNA, being circular, smaller than nuclear DNA and present in multiple copies, can be more resistant to degradation than nuclear DNA, in this way favoring the amplification of larger fragments.

### 2.2. Primer Design and Evaluation

The universal primers were used to evaluate the results of the DNA extraction, but they cannot be used to differentiate among the species. In fact, by using these primers, all plant species usually provide amplification, because they are developed on highly conserved sites of the gene among the different plant species. Despite this, these amplified fragments, while always present, can have sequence variations among the different species under investigation useful to develop more selective markers. To do this, the amplicons of the two plastid genes, *matK* and *rbcL*, belonging to the six different species of interest (saffron, curcuma, calendula, gardenia, safflower and buddleia) were sequenced and aligned, and the polymorphic sites were considered to develop the selective markers for the object species of the present study (Table 2). All the sequences, obtained in the present work, have been reported as Supplementary Materials.

**Figure 1:**
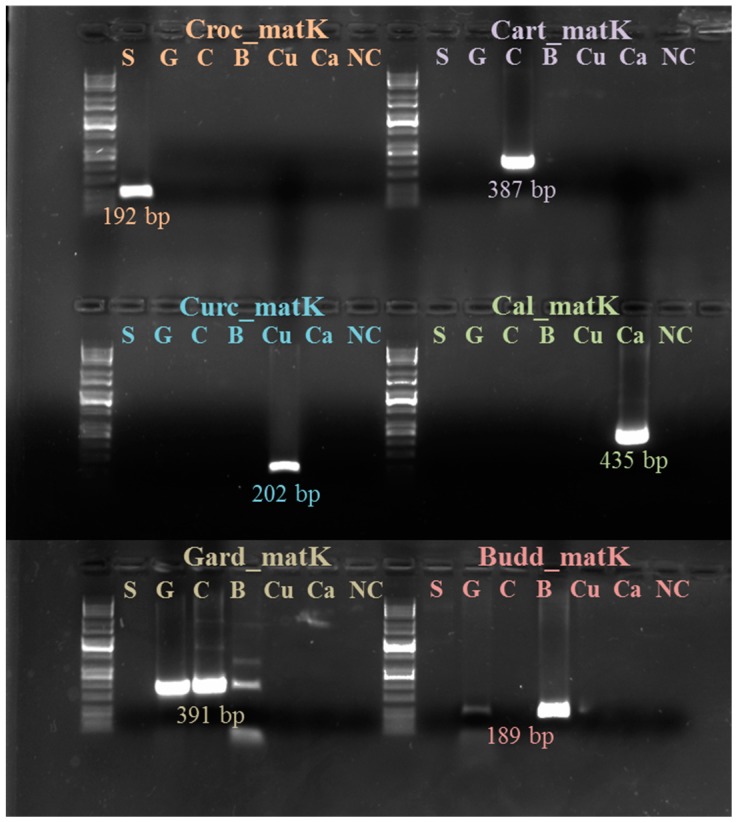
PCR amplification carried out with selective markers on: crocus (Croc), safflower (Cart), curcuma (Curc), calendula (Cal), gardenia (Gard) and buddleia (Budd). It is possible to see the high specificity of crocus, curcuma, safflower and calendula markers, while those for gardenia and buddleia also amplify in other species. For traceability purposes, it is important that the primers for the adulterants do not amplify in saffron and *vice versa*. Gardenia and buddleia were run on a separate gel, as clearly visible in the figure. Key: S (saffron), G (gardenia), C (safflower), B (buddleia), Cu (curcuma) and Ca (calendula).

We show evidence that among the *matK* sequences of the six species, there were more nucleotide variations than among the *rbcL* sequences. From Table 4 and Table 5, it is possible to notice that the percentage of sequence identity among the different *matK* sequences is smaller than the percentage among the different *rbcL* sequences (Table 4). The sequence identity for *matK* comprised between 73% (pairwise comparison between *C. sativus* and *G. jasminoides*) and 93% (*C. tinctorius* and *C. officinalis*), with an average value of 79.4%. The sequence identity for *rbcL* was between 89% (*C. sativus* with *C tinctorius* and *C. officinalis*) and 98% (*C. tinctorius* with *C. officinalis*), with an average value of 92.2%. Concerning the gaps among the sequences, for *rbcL,* the percentage is usually 0%, while among *matK* sequences, the same percentage is between 0% and 3%. These comparisons provided the clear indication that the nucleotide variation among *matK* sequences was bigger than among *rbcL* sequences. Because of this, it was possible to base the matK primers on a larger number of nucleotide variations than the selective primers on *rbcL*, and consequently, we could also expect greater selectivity for these. The specificity of the primers was tested on the DNA of the different species, and the results are reported in Table 5 and Figure 1.

Four matK markers out of six (more specifically, the markers for saffron, curcuma, safflower and calendula) were highly selective and amplified only in the expected species. The other two markers (the ones for buddleia and gardenia) showed less specificity, but none of them amplified products from saffron DNA: the marker for buddleia amplifies also in gardenia, and the marker for gardenia amplifies also in buddleia and, to some extent, in safflower. Despite this lower specificity, the absence of amplification in saffron makes it possible to also consider these two markers for traceability. It is important to note that the selectivity we are discussing is with respect to the species under investigation. In fact, as a result of database searching, it was possible to see that the marker developed on saffron *matK* is also able to amplify a high number of the crocus species belonging to the crocus series. This is a consequence of the fact that between the *matK* sequence of the crocus series, the number of nucleotide variations is very low, making it challenging to develop species-specific markers based on this region (although the chance of spice adulteration with other species of crocus stigmas, which would be even more difficult to grow than saffron, is low). The same can be possible concerning the markers for the other species.

Contrary to *matK*, the situation for the markers developed on RUBISCO was not so clear, but to trace the presence of an adulterant, it is important that the primers for the adulterant must not amplify in saffron and that the primers for saffron must not amplify in the adulterants. For this reason, the markers on *matK* were more specific and robust for our purposes, so we focused our attention just on these (Table 3 and Table 4). The matK-based markers have been tested on all of the samples of the list reported in Table 1. We obtained amplification for all of the samples, with the only exception represented, as expected, by the five samples corresponding to the solid and liquid buddleia and gardenia extracts.

Four samples, AD.01.JM, AD.02.JM, AD.12.JM and AD.13.JM, were provided as adulterated samples, but the only marker that amplified in these samples was the one specific for crocus, while none of the other markers provided any amplification.

**Figure 2:**
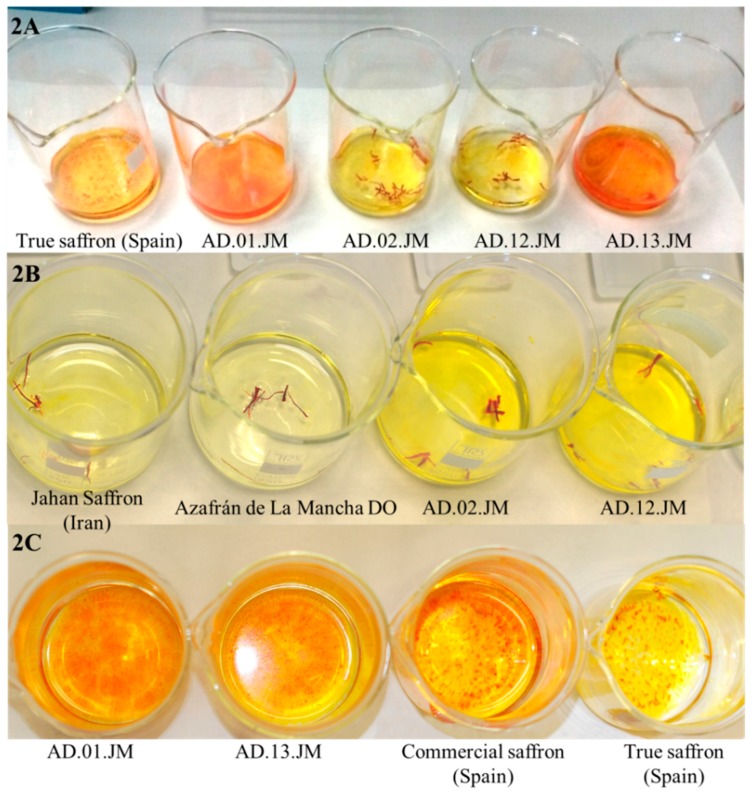
Comparison of the different coloring properties of true and adulterated saffron samples. (**A**) Different staining capacity of the same amount of true saffron powder (true saffron from Spain) compared to adulterated powders and stigmas. The color of true saffron is clearly less intense than the color of the two adulterated powders (AD.01.JM and AD.13.JM) and more or less similar to the color of the adulterated stigmas. The coloring properties of powder are stronger than the properties of stigmas. The fact that the color intensity of true saffron and stigmas is similar is a consequence of the adulteration of stigmas; (**B**) Different staining capacity of the same amount of true saffron (Jahan saffron, from Iran, and Azafrán de La Mancha DO (Denominación de origen), from Spain) and adulterated saffron (AD.02.JM and AD.12.JM) stigmas. It is evident that true saffron has less staining capacity than adulterated saffron in cold water. The latter samples probably release some synthetic dye; (**C**) Different staining capacity of the same amount of true saffron powder (true saffron from Spain), a commercial sample (commercial saffron from Spain) and the two adulterated samples used in this work. At the same time, adulterated and commercial samples produced a stronger coloration of water than true saffron.

**Figure 3:**
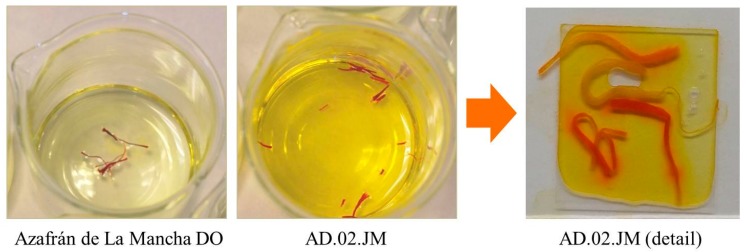
Comparison between same amounts of true saffron stigmas (Azafrán de La Mancha DO, Spain) and adulterated stigmas (Sample AD.02.JM) after just 3 min in cold water. It is clearly visible that, at the same time, adulterated saffron (dark yellow) releases color faster than true saffron (pale yellow). On the right, a detail of the adulterated stigmas from Sample AD.02.JM. There are clearly visible stigmas with different colors: red, pale orange, likely brown stigmas still decoloring and pale brown. The adulteration probably corresponds to the mixture and staining of two or more very old saffron stigmas with some fresh stigma lots.

In order to define the possible adulteration, we compared the behavior of these samples and of true saffron in releasing color in water. In Figure 2 and Figure 3, it is visible that the adulterated samples produced more intense color and that, at least in sample AD.02.JM, after soaking in water, there were stigmas with different colors (red and brown). We can hypothesize that the adulteration for these samples is the mixture of many different saffron of different years (stigmas become brown after several years) that have been stained by adding a synthetic dye, and this supports why we did not have amplification with other markers, except the one for crocus.

Based on these results, we can conclude that fraud carried out by using extracts or synthetic dyes cannot be detected using a DNA-based approach, but with inexpensive and readily-available spectroscopic methods or HNMR metabolite fingerprinting; see [6].

**Figure 4:**
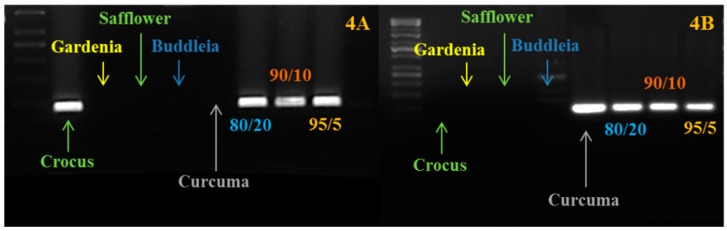
PCR amplification carried out using saffron and curcuma markers on saffron-curcuma artificial mixtures. (**A**) We show the amplification carried out using the crocus-specific marker; (**B**) We show the amplification carried out using the curcuma-specific marker. As expected, the amplification is present just in crocus, curcuma and in the mixtures. The ratio (such an 80/20) refers to the different percentages of saffron and curcuma powders mixed before the DNA extraction.

In order to be used for traceability, the developed markers must be validated in mixtures of plant species, showing the ability to recognize the presence of adulterants (even in very low percentages) within saffron samples, although it is true that real adulteration will come with the addition of significant amounts of contaminants. To do this validation to estimate the technique’s sensitivity, we prepared some artificially-adulterated samples: saffron + curcuma; saffron + buddleia; saffron + gardenia; saffron + safflower; and saffron + calendula. The mixes, saffron/adulterant, were prepared before the extraction of DNA with the following percentages of powders: 50/50; 80/20; 90/10; 95/5; 98/2; 99/1; 99.5/0.5. We were able to detect the presence of the adulterant DNA even at the lowest percentage (Figure 4). It is important to clarify that in this case, the level of adulteration refers to the amount of adulterant added before the extraction and not to the amount of adulterant DNA in the DNA extraction. It is recognizable that the ratio of the two DNAs (saffron and adulterant) in the final extraction may be different from the initial ratio between saffron and adulterant powder. Considering a mixture with just 0.5 µg of adulterant and 99.5 µg of saffron, the DNA ratio will be clearly unbalanced *versus* saffron DNA, which will be much higher than the adulterant DNA. As reported in other papers [8], we confirmed that DNA techniques have the ability to detect the presence of very low amounts of extraneous DNA. According to the ISO 3632 for saffron, for saffron belonging to Categories 1 and 2, the level of unwanted contaminations with material from other plants is respectively 0.1% and 0.5%. With these markers, we can detect the presence of, at least, 0.5% of adulterant. It is important to distinguish between adulteration and unwanted contamination. We think that because of this high sensitivity, DNA-based techniques must be applied with different steps: (1) qualitative PCR to confirm the presence of extraneous plant material; and (2) in positive samples, trying to quantify the relative amount of extraneous DNA by quantitative approaches.

### 2.3. AFLP and MS-AFLP Analysis of the Different Saffron Flower Parts

**Figure 5:**
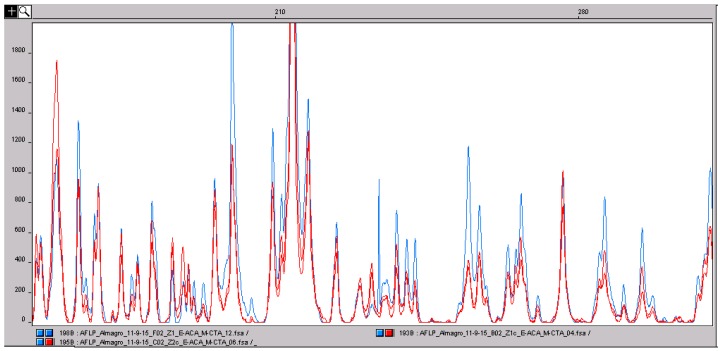
Comparison of the amplified fragment length polymorphism (AFLP) profile of saffron stigmas (blue) and two saffron powder samples (red). The peaks correspond to DNA fragments with a defined size in base pairs. The three profiles are almost perfectly superimposable. Usually, food processing can induce DNA degradation and, consequently, a change in the genetic profile obtained with multilocus molecular markers, such as AFLP. In this situation, the results clearly shows that the degradation of saffron DNA does not influence the AFLP genetic profile. This confirms the suitability of multilocus marker approaches to saffron analysis.

Saffron is one of the main adulterants of saffron itself. The addition of different parts of saffron flowers (tepals and stamens cut in pieces and colored) is one of the most widespread adulterations carried out by cheaters, mainly in the powder form. In fact, in whole stigmas, a simple microscopic analysis carried out by experts is usually enough to detect the presence of plant material of different origin. While the identification of different species can be easily achieved by developing informative primers, the situation is different when the adulterant is part of the same plant and has the same genetic profile. Further, in saffron, because the presence of only little genetic diversity [16,17], there are no different characterized cultivars as in other crops, and detecting adulteration of stigmas with other parts of the flower is not as simple to achieve by using molecular approaches. In a previous paper [18], we evidenced that, while genetic variation is low, epigenetic variation at the cytosine level is high between leaves sampled from accessions of different origin. Epigenetic changes, other than the geographic origin, can also be associated with the different parts of the same organism. Considering this, we decided to analyze and compare the genetic (AFLP) and the epigenetic (MS-AFLP) profiles of the different parts of the crocus flower. Because processing induces the degradation of DNA, the banding pattern of multilocus markers, such as AFLP and MS-AFLP, can change. To detect adulteration with other tissues of the same species, the starting point is to verify that DNA degradation does not change the banding pattern. To this aim, we carried out a preliminary analysis on three pure saffron samples, one made by stigmas and the other two in powder. As shown in Figure 5, the AFLP profiles of whole stigmas and powder are almost exactly the same. This means grinding does not determine DNA degradation so high so as to change the AFLP profile to a large degree, supporting the application of this technique for saffron authenticity.

**Figure 6:**
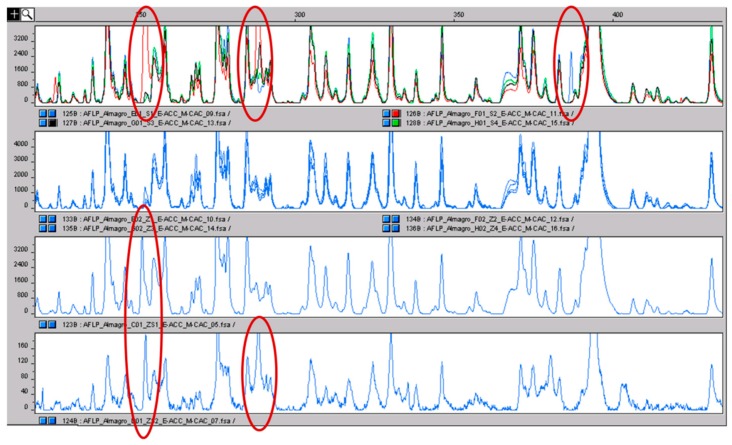
In the upper panel, the AFLP profiles of the four pools of stamens are shown in different colors. The polymorphic peaks are clear and evidenced with the red circle. The second panel shows the profiles of the four pools of stigmas coming from the same accessions from which the stamens were sampled: the color is equal because no inter-pool differences were present. In the last two panels, the profiles of two stigma-stamen mixtures are shown. The polymorphisms of the stamens, absent in stigmas, can be detected (red circles). The fact that the same polymorphisms were detected by repeating the analysis is a clear indication that they are true polymorphisms and not just false positives.

For these analyses, pools of stigmas, stamens and tepals were considered. The pools were made by mixing tissues from accessions with different geographic origin stored in the germplasm of Cuenca. The AFLP profiles of stigmas and tepals were exactly identical without any variation among pools or tissues. On the contrary, while very similar, some polymorphisms have been detected among the pools of stamens and between stamens and the other two tissues. To confirm the true nature of the polymorphic signals and to exclude the possibility that they are artefacts of the method, we repeated the same analyses in mixtures of tissues, 50% stigmas and 50% stamens, prepared before the DNA extraction. Repeating DNA extraction and AFLP analysis, we also found the same polymorphism in the mixtures (Figure 6). By using four selective primer combinations, a few genetic polymorphisms were detected, always in stamens, showing the presence of little genetic variability among different tissues. These analyses confirm that the genetic variability in saffron crocus is very low, and because of this, genetic variability is likely to be too low to be considered for detecting the contamination of stigmas with stamens; but, it may be worthy of further consideration.

**Figure 7:**
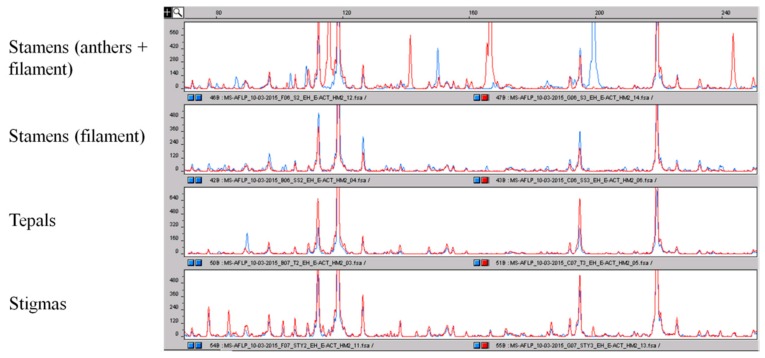
Comparison of the epigenetic profile obtained by using the MS-AFLP markers of the different parts of the crocus flower. As for AFLP, the different peaks correspond to DNA fragments of different sizes. It is clearly visible that the epigenetic profile of the whole stamens is very different with respect to the epigenetic profile of filaments and other parts of the flower that are more conserved. The polymorphisms among stamens and the other parts of the flower are highlighted.

The methyl-sensitive analysis provided more interesting results. Considering the MS-AFLP profile of stigmas as a reference, we noted that only very small differences were present in the epigenetic profile among the stigma pools, among the tepals pools and between stigmas and tepals. Contrary to what we noted in a previous work [18], where, working on leaves, we detected high epigenetic variation between single samples with different geographic origin, this time, using the pools of accessions, the epigenetic profiles were very similar. Possible explanations of these differences are: (1) mixing of material from different accessions with different geographic origin in making the pools; (2) working with different tissues, high variability was detected in leaves [18], while this time, we are working with the parts of flower. While genetic analysis, as expected, failed to detect any difference between these two tissues, with epigenetic analysis, a certain number of polymorphisms were detected. Despite this, the most interesting results were obtained with stamens. Stamens, the pollen-producing organ of the flower, is made by a filament and by an anther that holds pollen granules; the epigenetic profiles of the whole stamens, anther + filament, were significantly different, showing a high number of polymorphisms, both within the four pools of this tissue and with respect to the profiles of stigmas and tepals (Figure 7). The analyses were replicated independently, and each pool always provided the same epigenetic profile. Trying to explain this result, we speculated that the big variations were mainly a consequence of the presence of the pollen granules inside the anthers. To verify this, we tried to repeat the analysis of the stamens by removing the anthers and keeping just the filament. As a consequence, the MS-AFLP profile of the filaments was very similar to the profile of the stigmas, with just a few differences (Figure 7). This supports the role of anthers, likely of pollen granules, in producing the incredibly high epigenetic variation we observed. Working with multilocus markers, it is important to verify the reproducibility of the observed DNA fragments pattern. To give further support to these results, the analyses were replicated, and all of the time, the same data were obtained: high similarity among stigmas, tepals and filament; high variation among the other tissues and whole stamen epigenetic profile.

These findings open up the intriguing possibility of using this large variability at the epigenetic level, possibly because of the presence of pollen granules in the anthers, to detect massive adulterations of stigmas with stamens: not by using single PCR-based markers, but focusing on the variation induced in the global epigenetic profile by the presence of a big amount of whole stamens. Understandably, we need to continue this work in order to obtain further indications. To test this on commercial saffron samples, recently, we have applied the same methodologies (AFLP and MS-AFLP) also to some samples acquired in a local mall. We prepared some artificially-adulterated samples, by mixing the commercial saffron powder and stamens at the same percentage as previously reported. After the DNA extraction, the analyses were carried out, and after that, we compared the genetic and epigenetic profiles of the commercial saffron and of the mixtures with the genetic and epigenetic profiles of stigmas and stamens that we obtained previously (data not shown). As expected, the genetic variability was always very low or absent. Again, more interesting results were obtained with the epigenetic analysis. On the one hand, these analyses confirm that the MS-AFLP profiles of the commercial saffron were almost identical, with very little variability, to the epigenetic profiles of the pools of stigmas from Cuenca. This result is significant; in fact, it is recognized that the epigenetic profile can be influenced by the environment, and so, it could be expected that saffron samples with different geographic origin can have different epigenetic profiles also at the level of stigmas. Obviously, the existence of high variability between the epigenetic profile of stigmas with different geographic origin could hinder the application of epigenetic analysis for the detection of contamination with stamens: if high variability is already present among stigmas, the variation introduced by the presence of stamens would pass unnoticed. Concerning this point, at the moment, we do not have such indication, and our results suggested that the epigenetic profiles of stigmas were very similar, independent of the origin of the samples. On the other hand, the epigenetic profile of the mixtures of stigma-stamen was characterized by the presence of extra polymorphisms with respect to the profile of stigmas alone. In our opinion, this is an interesting possibility, and if these approaches could be useful or not in saffron traceability can be a matter of further investigations.

## 3. Experimental Section

### 3.1. Sample Set

The samples considered for the development of the informative molecular markers are listed in Table 1. Inside the sample set, two liquid and three solid (powder or lyophilized) extracts, putatively obtained from gardenia and buddleia, and purchased online, were reflected, as well. In order to validate the functionality of the developed markers, artificially-adulterated samples were prepared in the laboratory by adding 50%, 20%, 10%, 5%, 2%, 1% and 0.5% of the adulterants to saffron before DNA extraction. Saffron was mixed with: curcuma, safflower, calendula, buddleia, gardenia, gardenia liquid extracts, gardenia solid extracts and buddleia solid extracts. Mixing the samples before the extraction simulates a more realistic situation than mixing DNA after the extraction.

In addition, for the analysis of MS-AFLP and AFLP profiles of the different parts of the saffron flower, twelve pools of tissues (four pools of tepals, four pools of stamens and four pools of stigmas) were recovered from the same saffron crocus accessions at the World Saffron and Crocus Collection located at the Bank of Plant Germplasm of Cuenca (Cuenca, Spain) [19]. After collection, the samples were immediately processed for DNA extraction. Mixtures of 50% stigmas, or saffron powder, with 50% stamens or tepals were made before the DNA extraction to see if the eventual polymorphic signals between the different tissues could also be detected also in blends.

### 3.2. DNA Extraction and PCR Amplificability

The extraction of DNA from pools of different flower parts for MS-AFLP and AFLP analysis was carried out by using the “GeneElute Plant Genomic DNA Miniprep Kit (Sigma-Aldrich, Saint Louis, MO, USA)”, following the manufacturer’s instructions.

Regarding the samples listed in Table 1 and the artificially-adulterated samples, three different kits were tested in order to find the best method for our aim, which was the recovery of PCR-grade DNA, especially from the processed samples: GeneElute Plant Genomic DNA Miniprep Kit (Sigma-Aldrich); Plant DNA Purification Kit (Canvax, Córdoba, Spain); and DNeasy Plant Mini Kit (Qiagen, Venlo, the Netherlands).

The extractions were carried out following the manufacturer’s instructions with just a few modifications: (1) for the liquid extracts, before proceeding with DNA extraction, we adopted a precipitation protocol [20] in order to obtain, if possible, a pellet from which to extract DNA; (2) usually, plant material and derived products can have high concentrations of secondary metabolites that hinder the recovery of DNA and/or the subsequent enzymatic reactions. One of the most reported solutions in the literature to avoid this problem is the addition of substances like PVP (polyvinylpyrrolidone) directly during the DNA extraction to facilitate the removal of some secondary metabolites, such as polyphenols [21]. This can increase the purity of the recovered DNA and the possibility to analyze it. Considering this, and based on previous observations carried out in our laboratory, before the extraction, we added 4% PVP to each sample.

The results of the DNA extractions in terms of quantity and quality were evaluated by agarose gel electrophoresis: 1% agarose for the DNA extracted from leaves and petals, 1.5% agarose for all the other samples, because we expected a more degraded DNA from processed matrices.

The amplificability of the extracted DNA and the size of the obtainable amplifiable fragments have been evaluated by using three universal primer pairs classically used for plant DNA barcoding. Barcoding involves the use of primer pairs developed on different nuclear and plastid DNA regions. In this study, we used two primer pairs developed on the plastid genes for RUBISCO large subunit (*rbcL*-F and *rbcL*-R) and maturase K (*matK*-KIM1R and *matK*-KIM1F) and a primer pair specific for the internal transcribed spacer regions (ITS-S2F and ITS4) of the nuclear genes for the large ribosomal RNA (Table 2); see [22]. PCRs were carried out in a final volume of 25 µL containing 10 ng or 1 µL (if the extracted DNA was not visible on agarose gel) of DNA template, 1X PCR buffer (Promega, Fitchburg, WI, USA), 1.5 mM Mg^2+^, 0.15 mM dNTPs, 1 µL of each primer 10 µM, 4% PVP and 1 U DNA Polymerase (Promega). The amplification cycle parameters were: 95 °C for 5 min, 35 cycles of 30 s at 95 °C, 40 s at the corresponding annealing temperature (Table 2), 1.5 min at 72 °C, and a final extension of 10 min at 72 °C. The addition of PVP was performed in order to improve the amplificability of the recovered DNA. The products of the PCR reactions were loaded and run on an agarose gel (1.5%, 0.5X TBE (Tris/Borate/EDTA)). It is reported in the literature that amplification inhibition because of polyphenols can be eliminated by adding PVP to the PCR mixture itself, making DNA more suitable for amplification [23].

### 3.3. Marker Development and Validation

The amplicons of the expected size obtained with the universal primers for chloroplast DNA were excised and sequenced as reported in the literature [24]. The sequences have been submitted to GenBank, and the accession numbers are reported in Table 4. Homology searches by the BLAST program and NCBI database were carried out to check the correspondence between the amplified fragments and the expected regions. After this, sequences were aligned, and using the polymorphic regions, more selective primers were designed for the different plant species under investigation (*C. sativus*, *C. tinctorius*, *C. longa*, *G. jasminoides*, *B. officinalis* and *C. officinalis*; Table 2). Pairwise comparisons to estimate the percentage of nucleotide identity and of gaps among the sequences were carried out by using the BLAST two sequences (bl2seq) tool [25] (Table 4).

The validation of these markers was carried out by PCR in a final volume of 25 µL containing a maximum of 10 ng or 1 µL of recovered DNA 1X PCR buffer (Promega), 1.5 mM Mg^2+^, 0.15 mM dNTPs, 10 pmol of each primers, 4% PVP and 1 U DNA Polymerase (Promega). The cycling parameters were: 95 °C for 5 min, 35 cycles of 30 s at 95 °C, 40 s at the corresponding annealing temperature (Table 2), 1 min at 72 °C and a final extension at 72 °C for 10 min. The PCR products were loaded and run on an agarose gel (2%, 0.5X TBE). The annealing temperature for each primer pair reported in Table 2 is the temperature optimized after different trials. The capacity of the markers to recognize the presence of adulteration or contamination was carried out by using the DNA recovered from the adulterated, or artificially-adulterated, samples.

### 3.4. AFLP and MS-AFLP Analysis of the Saffron Flower Parts

The AFLPs and the methyl-sensitive AFLPs were carried out as reported in [18]. The amount of enzymes to use was evaluated by using the double digest application available on the Fermentas website. The AFLP technique was carried out by using the restriction enzymes *Eco*RI and *Mse*I. The MS-AFLP technique used *Eco*RI and alternatively the isoschizomer pair *MspI* and *HpaII,* whose ability to cleave at 5′-CCGG-3′ is heavily affected by the methylation state of the cytosines. *HpaII* is inactive if one or both cytosines are fully methylated (both strands methylated), but cleaves the hemi-methylated sequence (only one strand methylated), while *MspI* cleaves C5mCGG, but not 5mCCGG. *Eco*RI is usually considered methylation-independent. The pre-selective amplifications have been carried out using the primer combinations E01/M02 for AFLP and E01/HM-T (E01 and M02 are the primers respectively designed on the *Eco*RI and *Mse*I adapters corresponding to the standard nomenclature for AFLP primers, while HM is the pre-selective primer specific for the HspI/MspII adapter, and T is the pre-selective nucleotide). The following selective primer combinations, reported using the standard nomenclature for AFLP primers, have been considered: E36/M48, E35/M49, E35/M50 and E35/M59 for classic AFLP and E38/HM-TCC, E40/HM-TAA and E32/HM-TTC for MS-AFLP. The Eco primers are the same generally used also for classic AFLP, while the HM primers have the following base sequence: 5′-ATCATGAGTCCTGCTCGG-3′. The selective EcoRI primers (for both AFLP and MS-AFLP analysis) were labelled with fluorescent dyes (6-FAM for primers E35 and E38; NED for primers E32 and E36; TET for primer E40). The amplified products from selective amplifications were loaded and run on the ABI Prism 3130 Genetic Analyser (Life Technologies) and analyzed using the software provided with the instrument.

## 4. Conclusions

For traceability purposes, the recovery of PCR-grade DNA from processed matrices is of fundamental importance. By comparing different DNA extraction kits, we demonstrated that the choice of kit can influence the results of the extraction and, in particular, the possibility of recovering PCR-grade DNA. While the pattern on agarose gel was practically identical for the three kits, the best amplifications were obtained with the “GeneElute Plant Genomic DNA” kit. The size of the amplicons obtained and the correspondence of the AFLP banding pattern between saffron stigmas and powder indicate that, for these matrices, processing and DNA degradation do not hinder the possibility of using or developing a DNA-based approach for traceability.

Using the recovered DNA, we were able to design some new selective markers that could be very useful in implementing the existing approaches for traceability. These markers, mainly the ones developed on the sequence of the plastid gene for the enzyme maturase K, were found to be very selective and able to discriminate the different adulterant species from saffron and to also detect the presence of very small amounts of extraneous DNA in artificially-prepared mixtures.

From the extracts, we were unable to recover any amplifiable DNA; the more likely explanation is that in these extracts, the DNA is completely degraded or absent. Considering this, adulteration with similar extracts cannot be detected by using DNA approaches, but we must rely on the existing chemical methods. We must say that we ignore how these extracts have been made. It could be interesting to have some other extracts obtained from plant material in order to reattempt the DNA extraction.

Finally, concerning the possibility of detecting the addition of different parts of the saffron flower, some interesting results have been observed by using the AFLP and, in particular, MS-AFLP markers. We evidenced a large difference in the marker profile of whole stamens (anthers and filaments) with respect to the same profile of stigmas and tepals. This opens up the interesting possibility of detecting one of the main adulterations in saffron production: the addition of stained stamens.

## Figures and Tables

**Table 1:** The samples considered for the setup of the DNA extraction methods and the development of the informative markers are listed. Visually, the three methods provided the same results in terms of amount and integrity of recovered DNA.

Name ^a^	Description	Origin	DNA Extraction ^b^
AD.01.JM	Saffron (*C. sativus*) powder adulterated	France	-
AD.02.JM	Saffron stigmas adulterated	France	-
AD.03.JM	Gardenia (*G. jasminoides*) extract (liquid)	France	n.v.
AD.04.POL	Safflower (*C. tinctorius*) petals	Greece	+
AD.05.POL	Gardenia fruit extract (powder)	Greece	n.v.
AD.06.POL	Calendula (*C. officinalis*) petals	Greece	+
AD.07.POL	Saffron stamens	Greece	--
AD.08.POL	Curcuma (*C longa*) rhizome powder	Greece	--
AD.09.POL	Buddleia (*B. officinalis*) powder extract	Greece	n.v.
AD.10.POL	Buddleia powder extract	Greece	n.v.
AD.11.JM	Curcuma powder	France	--
AD.12.JM	Saffron stigmas adulterated	France	-
AD.13.JM	Saffron powder adulterated	France	-
AD.14.JM	Gardenia extract (liquid)	France	n.v.
AD.15.BM	Curcuma powder	Italy	--
AD.16.BM	Saffron powder Commercial A	Italy	-
AD.17.BM	Saffron powder Commercial B	Italy	-
AD.18.BM	Saffron leaves	Italy	+
AD.19.BM	Safflower leaves	Italy	+
AD.20.BM	Gardenia leaves	Italy	+
AD.21.BM	Buddleia leaves	Italy	+
AD.22.JM	Gardenia fruits	France	n.v.

^a^ AD: ADulteration project; JM: Jean-Marie; POL: POLissiou; BM: Busconi Matteo. ^b^ Visible signal on agarose gel. Samples marked with “+” were usually characterized by high quality DNA; “-” or “--” samples were usually characterized by DNA degradation, as supported by the presence of a more (-) or a less (--) intense smear; and samples with “n.v.” were characterized by the absence of any visible (no visible DNA) signal.

**Table 2:** Details of species-specific primer pairs used for the amplifications in this study. Universal primers are divided from the primers developed in the present study with a line.

Primer Name ^a^	Sequence	Annealing Temperature	Amplicon Size (bp)	Typology
matK*-*KIM1R	ACCCAGTCCATCTGGAAATCTTGGTTC	58 °C	Variable, *circa* 900	Universal
matK-KIM3F	CGTACAGTACTTTTGTGTTTACGAG			
rbcL-F	ACCACAAACAGAGACTAAAGC	52 °C	Variable *circa* 600	
rbcL-R	GTAAAATCAAGTCCACCRCG			
ITS-S2F	ATGCGATACTTGGTGTGAAT	52 °C	Variable *circa* 400	
ITS4	TCCTCCGCTTATTGATATGC			
Gard_matK_Fw	TGGGATACTCTTATTGATAG	55 °C	391	Primers developed in the present study
Gard_matK_Rev	CCGGGTGAAACCAAATAC			
Budd_matK_Fw	GAACGTCTTTGTTAAGGTTAAG	58 °C	189	
Budd_matK_Rev	CTTGGATGAAACCAAAGCGA			
Curc_matK_Fw	GTAAAAATAGAACATCTTGGAG	56 °C	202	
Curc_matK_Rev	ATATGGTTGAGACCAAAAATG			
Cart_matK_Fw	TGTATGTGAATATGAATCTGGC	54 °C	387	
Cart_matK_Rev	CCATTGAACGCTTTACCGCG			
Croc_matK_Fw	ATCTTATAATAGTATGTTGTGAT	54 °C	192	
Croc_matK_Rev	TGTATGATTGATACCAAAAGT			
Cal_matK_Fw	CATACTCTGGGCCACAAC	53 °C	435	
Cal_matK_Rev	GAGGAAGCCGTATTCATATT			

^a^ Gard, gardenia; Budd, buddleia; Curc, curcuma; Cart, *Carthamus*; Croc, crocus; Cal, calendula.

**Table 3:** Results of the amplificability tests with the universal primers for plant barcoding on the extractions carried out with the three methods.

Sample	Extraction Methods								
	GeneElute Plant			Plant DNA Purification			DNeasy Plant		
	matK	rbcL	ITS	matK	rbcL	ITS	matK	rbcL	ITS
	>900 ^a^	>600	>400	>900	>600	>400	>900	>600	>400
AD.01.JM	+ ^b^	+	+	/	/	/	/	/	/
AD.02.JM	+	+	+	/	/	/	/	/	/
AD.03.JM	/	/	/	/	/	/	/	/	/
AD.04.POL	+	+	+	+	+	+	+	+	+
AD.05.POL	/	/	/	/	/	/	/	/	/
AD.06.POL	+	+	+	+	+	+	+	+	+
AD.07.POL	+	+	+	/	/	/	+/−	+/−	+/−
AD.08.POL	+	+	+	/	/	/	/	/	/
AD.09.POL	/	/	/	/	/	/	/	/	/
AD.10.POL	/	/	/	/	/	/	/	/	/
AD.11.JM	+	+	+	/	/	/	/	/	/
AD.12.JM	+	+	+	/	/	/	/	/	/
AD.13.JM	+	+	+	/	/	/	/	/	/
AD.14.JM	/	/	/	/	/	/	/	/	/
AD.15.BM	+	+	+	/	/	/	/	/	/
AD.16.BM	+	+	+	/	/	/	/	/	/
AD.17.BM	+	+	+	/	/	/	/	/	/
AD.18.BM	+	+	+	+	+	+	+	+	+
AD.19.BM	+	+	+	+	+	+	+	+	+
AD.20.BM	+	+	+	+	+	+	+	+	+
AD.21.BM	+	+	+	+	+	+	+	+	+
AD.22.JM	+	+	+	/	/	/	/	/	/

^a^ expected size of the corresponding amplicons in base pairs; the expected size is higher than: 900 bp for matK; 600 bp for rbcL and 400 bp for the internal transcribed spacer (ITS). ^b^ a positive result or the absence of amplification are reported respectively with “+” and “/”. +/− refers to samples with a very weak amplification.

**Table 4:** Pairwise comparison of the tbcL, under the diagonal, and *matK*, above the diagonal, sequences for the different species. Percentages of nucleotide identity and gaps are reported. The accession numbers for the sequences are: crocus, KU230342 (*rbcL*) and KU230351 (*matK*); curcuma, KU230346 (*rbcL*) and KU230349 (*matK*); buddleia, KU230343 (*rbcL*) and KU230348 (*matK*); gardenia, KU230345 (*rbcL*) and KU230347 (*matK*); safflower, KU230344 (*rbcL*) and KU230350 (*matK*). For calendula, we considered the sequences available online in GenBank with Accession Numbers KM356099 (*rbcL*) and AF151446 (*matK*).

rbcL	*Crocus sativus*		*Gardenia jasminoides*		*Buddleja officinalis*		*Curcuma longa*		*Carthamus tinctorius*		*Calendula officinalis*		matK
	Id%	gap%	Id%	gap%	Id%	gap%	Id%	gap%	Id%	gap%	Id%	gap%	
*Crocus sativus*	/	/	73	3	74	2	80	2	75	2	74	2	*Crocus sativus*
*Gardenia jasminoides*	91	0	/	/	74	2	76	1	83	1	83	1	*Gardenia jasminoides*
*Buddleja officinalis*	90	0	95	0	/	/	76	1	85	1	82	1	*Buddleja officinalis*
*Curcuma longa*	94	0	90	0	90	1	/	/	76	1	74	2	*Curcuma longa*
*Carthamus tinctorius*	89	0	95	0	94	0	90	0	/	/	93	0	*Carthamus tinctorius*
*Calendula officinalis*	89	0	94	0	94	0	90	0	98	0	/	/	*Calendula officinalis*

**Table 5:** Specificity of the amplification carried out with the selective primers developed in the project.

matK Markers	Saffron	Safflower	Curcuma	Buddleia	Gardenia	Calendula
Croc_matK	+					
Cart_matK		+				
Curc_matK			+			
Gard_matK		+		+	+	
Budd_matK				+	+	
Cal_matK						+

## Supplementary Materials

*matK* and *rbcL* sequences are available online at: http://www.mdpi.com/1420-3049/21/3/343/s1.
