# Supplementary material for: Genetic and Epigenetic Approaches for the Possible Detection of Adulteration and Auto-Adulteration in Saffron (Crocus sativus L.) Spice
Source: Molecules. 2016 Mar 11;21(3):343. doi: 10.3390/molecules21030343 (PMC6273936; doi:10.3390/molecules21030343)
Supplement: Supplementary file 1 [file molecules-21-00343-s001.pdf]

Supplementary Materials: Genetic and Epigenetic Approaches for the Possible Detection of Adulteration and Auto-Adulteration in Saffron (*Crocus sativus* L.) Spice

Giovanna Soffritti , Matteo Busconi , Rosa Ana Sánchez , Jean-Marie Thiercelin , Moschos Polissiou , Marta Roldán and José Antonio Fernández

Maturase K sequences

>Gardenia jasminoides plastid gene maturase K (matK), partial cds;

ACCCAGTCCATCTGGAAATCTTGGTTCAAAACCTTCGCTATTGGGTAAAAGACGCCTCTTCC
TTGCATTTATTACGATTCTTTTTCCACGAATATTGGAATTGGGATACTCTTATTGATAGAAAG
AAACCCAGTTTTTATTTTTCACCAAAAAGAAATCAAAAATTATTCTTCTTGTTATATAATTCT
CATGTATGTGAATACGAATCCATTTTCTTCTTTCTCCGTAAGGAATCTTCTCATTTGCGATCA
ACATCTTTTGGAGTCTTTCTTGAACGGATATATTTCTATGAAAAAAAAGAACGTCTTGTAGA
AGTCTTTGCTAAGGATTTTCAAGCCAGTATATGGTTGTTCAAAGATCCTTTCATACATTATGT
TAGGTATCAAGGAAAATCCATTCTGGTTTCAAATGGGACGCCTCTTTTGATGAATAAATGG
AAATCTTACCTTGTCAATTTTTGGCAATGTCATTTTGGTATTTGGTTTCACCCGGGAAGGGTC
TATATAAACCAATTACCCAACTATTCCCTTAACTTTATGGGCTATCTGTCAAGTGTGCGACT
AAACCATTCAATGGTACGGAGTCAAATGCTAGAAAATTCATTTCTAATCAATAATGCTATT
AAGAAATTTGATACCCTTGTTCCACTTATTCCCCTCATTGGATCATTGGCTAAAGCGAAATT
TTGTAACTTATTAGGACATCCCATTAGTAAGCCGATTTGGACTGATTTGGCAGATTCTGATA
TTATTGACCGATTTTTGCATATATGCAAAAACCTTTCTCATTATTATAGCGGATCTTCCAAAA
AAAAGAGTTTGTATCGAATAAAGTATATACTTCGACTTTCTTGTGCTAAAACTTTGGCTCGT
AAACACAAAAGTACTGTACG

>Buddleja officinalis plastid gene maturase K (matK), partial cds;

ACCCTGTCCATGCAGAAATCTTGGTTCAAACTCTTCGCTATTGGGTAAAAGATGCCTCTTCT
TTGCATTTATTACGATTCTTTCTCAACGAGTCTTGTAATTGGAATAGTCTTATTACTCCAAAG
AAAGCCAGTTCCTCTTTTTCAAAAAGAAATCAAAGATTATTCTTATTCTTATATAATTCTCAT
GTATGTGAATACGAATCCATTTTCGTCTTTCTACGTAATCAATCTTCTCATTTAAGATCAACA
TCTTCTGGAGTTTTTCTTGAACGAATCTATTTCTATGGAAAAATAGAACGTCTTGTGAACGTC
TTTGTTAAGGTTAAGGATTTTCAGGAGAACCTATGGTTGGTCAAGGAACCTTGCATGCATTA
TGTTAGGTATCAAAGCAAATCCATTCTGGCTTCAAAAGGGACGTCTCTTTTAATGAATAAAT
GGAAATGGTACCTTGTCACTTTTTGGCAATGGTATTTTTCGCTTTGGTTTCATCCAAGAAAGA
TTTATATAAACCAATTATCCAATCATTCCCTTGAATTTTTGGGCTATCTTTCAAGCGTGCGAA
TGAACCCTTCAGTGGTACGGAGTCAAATTCTAGAAAATTCATTTCTAATCAATAATGCTATT
AAGAAGTTCGATACTCTTGTTCCAATTATTCCGCTGATTGCGTCATTGGCTAAAGCGAAATT
TTGTAACGTATTAGGGCATCCCATAAGTAAGCCGGTTTGGGCTGATTTATCAGATTCTAATA
TTATTGATCGATTTGGGCGTATATGCAGAAACCTTTCTCATTATCATAGCGGATCTTCCAAA
AAAAAGAGTTTGTATCGAATAAAGTATATACTTCGACTTTCTTGTGCTAGAACTTTGGCTCG
TAAACACAAAAGTACTGTACG

>Curcuma longa plastid gene maturase K (matK), partial cds;

ACCCTGTCCATGCAGAAATCTTGGTTCAAATGCTTCAATCCTGGATCCAGGATGTTCTCTCTT
TACATTTATTGCAGTTCCTTCTCCACGAATATTATAATTGGACTAGTCTCATTATTCCGAAAA
AATCTATTTACGTATTTTCAAAAGAAAATAAAAGACTATTTTGGTTCTTATATAATTTATATA
TATATGAATACGAATTTCTATTAGTGTTTCCTTGTAAACAATCCTCTTTTTTACGATTAATATC
TTCTGGAGTCCTTCTTGAGCGAATACATTTTTATGTAAAAATAGAACATCTTGGAGTGTGCC

GAATTTTTTGTCAGAAGACTCTATGGATTTTCAAGGATCCTTTCATACATTATATTCGATATC
AAGGAAAATCGATTCTGGGTTCAAGAGGGACTCATTTTTTGATGAAGAAATGGAAATACCA
CCTTGTTAATTTTTGGCAATATTATTTTCATTTTTGGTCTCAACCATATAGGATTGATATAAA
GAAATTATCAAACTATTCTTTTTATTTTCTGGGTTATTTTTCAAGTGTACAAATTAATTCTTCG
ATGGTAAGGAATCAAATGCTAGAGAATTCATTTCTGATGGATACTCTTACTAAGAAATTTG
ATACCATAATCCCAATTATTCCTCTTATTCGATCATTGTCTAAAGCTCAATTTTGTACCGTAT
CTGGGTATCCTATTAGTAAACCAATTTGGACCGATTTAGCGGATTGTGATATTATTAATAGA
TTTGGTCGGATATGTAGAAAGCTTTCTCACTATCACAGTGGATCCTCAAAAAAACAGAGTTT
GTATCGAATGAAGTATATACTTCGACTTTCATGTGCCAGAACTTTGGCTCGTAAACACAAA
AGTACTGTACG

>Carthamus tinctorius plastid gene maturase K (matK), partial cds;

ACCCTGTCCATGCAGAAATCTTGGTTCAGGCTCTTCGCTATTGGATAAAAGATGCTTCTTCTT
TGCATTTATTAAGATTCTTTCTCTATGAGTGTCATAATTGGGATAGTCTTATTACTTCAAATT
CAAAGAAAGCCAGTTCTTCTTTTTCAAAAAGAAATCACAGACTATTCTTTTTCCTATATACT
TCTCATGTATGTGAATATGAATCTGGCTTCATCTTTCTCCGTAACCAATCTTCTCACTTACGA
TCAACATCTTCTGGAGCCCTTCTTGAACGAATATATTTCTATGGAAAAATGGAGCATCTTGC
AAAAGCCTTTGCCAGGGCTTTTCAAGCTAATTTATGGTTGTTCAAAGATCCTTTCATGCATT
ATGTTAGGTATCAAGGAAAATCAATTCTTGCTTCAAAAGGGACGTTTCTTTTGATGAATAAA
TGGAAATATTACTTTGTCAATTTCTGGAAATCTTATTTTTACCTGTGGTCTGAACCAGGAAG
GATTTATGTAAACCAATTATCCAACCATTCCCTTGACTTTCTGGGTTATTGTTCAAGTGTGCG
GCTAAAGCGTTCAATGGTCCGCAGTCAAATGCTAGAAAATGCATTTCTAATCGATAATGCT
ATTAATAAATTTGATACTATTGTTCCAATTATGCCTCTGATTGGATCCTTGGCTAAATCTAAA
TTTTGTAACGCATTGGGGCATCCTATCGGTAAGGTGATTTGGGCCAATTTATCAGATTCTGA
TATTATTGATCGCTTTGGGCGTATATACAGAAACCTTTCTCATTATCATAGTGGATCTTCAAA
AAAAAAGAGTTTGTATCGAGTAAAGTATATACTTCGACTTTCTTGTGCTAGAACTTTAGCTC
GTAAACACAAAAGTACTGTACG

>Crocus sativus plastid gene maturase K (matK), partial cds;

ACCCTGTCCATGCAGAAATCTTGGTTCAAATCCTTCAATGCTGGATTCAAGATGTTCCTCTTT
TGCATTTCTTGCGATTCTTTCTTCACGAATATCATAATTGGAATAGTTTTTTAATTACTCAGA
ATAAATCTATTTATCTTTTTTCAAAAGAAACTAAAAGACTATTTCGGTTCCTATACAATTCTT
ATGTATATGAATGTGAATTTGTATTCGTTTTTCTTCGTAAGCACTCTTCTTATTTACGATTCAC
ATCCTTTCAAACTTTTCTTGAGCGAAGATATTTCTATGGAAAAATGGAACATCTTCAAACAG
AACATCTTATAATAGTATGTTGTGATTATTTTAATGGAACCCTATGGTCCTTCAAAGATCCTT
TCATGCATTATGCTCGATGTCAAGGAAAAGCAATTCTGGCTTCAAAAGGAACTCATCTTCTG
ATGAAGAAATGGAAATATAATTTTGTCAATTTATGGCAAAATTATTTTTACTTTTGGTATCA
ATCATACAGGATCCATATAAACCAACTATCAAACCATTCTTTCCATTTTCTGGGTTATCTTTC
AAGTTTACTAAAAAAATTCTTCGACGGTAAGGAATCAAATGTTAGATAATTCATTTCTAATAG
ATACTCTTACTATGAAATTTGATACCGCAGTCCCAGTTATTTTTCTTATTAGATCTTTATCTA
AAGCTCAATTTTGTACCGTATCGGGACATCCTATTAGTAAGCCCATCTGGACCGATTTATCA
GATTCTAGTATTATTGAGCGATTTGGTCGGATATGTAGAAATCTTTCTCATTATCATAGTGGG
TCCTCAAAAAAACAGGGGTTGTATCGAATAAAG

RUBISCO large subunit sequences

>Crocus sativus plastid gene RUBISCO large subunit (rbcL), partial cds;

ACCACAAACAGAGACTAAAGCAAGTGCTGGATTTAAGGCTGGTGTTAAAGATTACAGATT
GACTTATTATACTCCGGATCACGAAACCAAAGATACTGATATCTTGGCAGCATTCCGAGTA
ACTCCTCAACCCGGAGTTCCTGCTGAAGAAGCGGGGGCAGCGGTAGCTGCGGAATCTTCTA
CTGGTACATGGACAACAGTGTGGACTGATGGACTTACCAGTCTTGATCGTTACAAAGGACG
ATGCTATCATATCGAGGCCGTTGTTGGGGAGGAAAATCAATATATTGCTTATGTAGCTTATC

CTTTAGACCTTTTTGAAGAAGGTTCTGTTACTAATATGTTTACTTCCATTGTGGGTAATGTAT
TTGGTTTCAAAGCCCTACGAGCTCTACGTCTGGAGGATTTGCGAATTCCCCCTGCTTATTCC
AAAACTTTCCAAGGCCCACCCCATGGCATCCAGGTTGAAAGAGATAAATTAAACAAGTAT
GGTCGTCCCCTATTGGGATGTACTATTAAACCAAAATTGGGATTATCTGCAAAAAACTACG
GTAGAGCGGTTTATGAATGTCTACGCGGTGGACTTGATTTTAC

>Buddleja officinalis plastid gene RUBISCO large subunit (rbcL), partial cds;

ACCACAAACAGAGACTAAAGCAAGTGTTGGATTCAAAGCGGGTGTTAAAGAGTACAAATT
GACTTATTATACTCCTGAATACGAAACCAAAGATACTGATATCTTGGCAGCATTCCGAGTA
ACTCCTCAACCTGGAGTTCCGCCTGAAGAAGCAGGGGCCGCGGTAGCTGCCGAATCTTCTA
CTGGTACATGGACAACTGTGTGGACCGATGGACTTACGAACCTTGATCGTTACAAAGGGCG
ATGCTACCACATCGAGCCGGTTCCTGGAGAAACAGATCAATATATCTGTTATGTAGCTTACC
CTTTAGACCTTTTTGAAGAAGGTTCTGTTACTAACATGTTTACTTCCATTGTAGGAAATGTAT
TTGGATTCAAAGCCCTGCGTGCTCTACGTCTGGAAGATCTGCGAATCCCTCCTGCTTATGTT
AAAACTTTCCAAGGCCCGCCTCATGGGATCCAAGTTGAGAGAGATAAATTGAACAAGTAT
GGTCGTCCCCTGTTGGGATGTACTATTAAACCTAAATTGGGGTTATCTGCTAAAAACTATGG
TAGAGCAGTTTATGAATGTCTTCGCGGTGGACTTGATTTTAC

>Carthamus tinctorius plastid gene RUBISCO large subunit (rbcL), partial cds;

ACCACAAACAGAGACTAAAGCAAGTGTTGGATTCAAAGCTGGTGTTAAAGATTATAAATT
GACTTATTATACTCCTGACTATAAAACCAAGGATACTGATATCTTGGCAGCATTTCGAGTAA
CTCCTCAACCTGGAGTTCCGCCTGAAGAAGCAGGGGCCGCAGTAGCTGCCGAATCTTCTAC
TGGTACATGGACAACTGTGTGGACCGATGGGCTTACGAGCCTTGATCGTTACAAAGGGCGA
TGCTATGGAATCGAGCCTGTTCCTGGAGAAGAAACTCAATTTATTGCTTATGTAGCTTACCC
ATTAGACCTTTTTGAAGAAGGTTCTGTTACTAACATGTTTACTTCCATTGTAGGTAATGTATT
TGGGTTCAAAGCCCTGCGTGCTCTACGTCTGGAAGATTTGCGAATCCCTACTGCGTATGTTA
AAACTTTCCAAGGTCCGCCTCACGGCATCCAAGTTGAGAGAGATAAATTGAACAAGTATGG
TCGTCCCCTGTTGGGATGTACTATTAAACCTAAATTGGGGTTATCCGCTAAAAACTACGGTA
GAGCTGTTTATGAATGTCTTCGCGGTGGACTTGATTTTAC

>Gardenia jasminoides plastid gene RUBISCO large subunit (rbcL), partial cds;

ACCACAAACAGAGACTAAAGCAAGTGTTGGATTCAAAGCTGGTGTTAAAGAGTACAAATT
GACTTATTATACTCCTGAATACGAAACCAAAGATACTGATATCTTGGCAGCATTCCGAGTA
ACTCCTCAACCGGGAGTTCCACCTGAAGAAGCGGGGGCCGCGGTAGCTGCCGAATCTTCTA
CTGGTACATGGACAACTGTGTGGACCGATGGGCTTACCAGCCTTGATCGTTACAAGGGTCG
ATGCTATCACATCGAGCCAGTTCCTGGAGAAGAAGATCAATTTATTGCTTATGTAGCTTACC
CCTTAGACCTTTTTGAAGAAGGTTCTGTTACTAACATGTTTACTTCCATTGTAGGTAATGTAT
TTGGGTTCAAAGCCCTGCGCGCTCTACGTCTGGAAGATTTGCGAATCCCTACTTCTTATGTT
AAAACCTTCCAAGGGCCGCCTCATGGCATCCAAGTTGAGAGAGATAAATTGAACAAGTAT
GGTCGTCCCCTGTTGGGATGTACTATTAAACCTAAATTAGGTTTATCTGCTAAAAACTACGG
TAGAGCTGTTTATGAATGTCTTCGCGGTGGACTTGATTTTAC

>Curcuma longa plastid gene RUBISCO large subunit (rbcL), partial cds;

ACCACAAACAGAGACTAAAGCAAGTGTTGGATTTAAAGCTGGTGTTAAAGATTACAAATT
GAATTATTATACTCCTGACTACGAAGTCAAAGATACTGATATCTTGGCAGCATTCCGAGTA
ACTCCTCAACCTGGAGTTCCACCCGAAGAAGCAGGGGCTGCGGTAGCTGCCGAATCTTCCA
CTGGTACATGGACAACTGTGTGGACTGATGGACTTACCAGTCTTGATCGTTACAAAGGGCG
ATGCTACCACATAGAGCCTGTTATTGGGGAGGATAATCAATATATTGCTTATGTAGCTTATC
CATTAGACCTTTTTGAAGAAGGTTCTGTTACTAACATGTTTACTTCCATTGTGGGTAATGTGT
TTGGTTTCAAAGCCTTACGCGCTCTACGTTTGGAGGATCTGCGAATTCCCACTTCCTATTCCA
AAACTTTCCAAGGCCCGCCTCACGGCATTCAGGTTGAAAGAGATAAGTTGAACAAGTATGG

TCGTCCCCTATTGGGATGTACTATTAAACCAAAATTGGGATTATCTGCAAAAAACTACGGT
AGAGCAGTTTATGAGTGTCTACGCGGTGGACTTGATTTTAC
